# Nanorobotic Approaches Against Multidrug-Resistant Infections: Design, Principle, Mechanistic Innovation, Translational Challenges and Biomedical Applications

**DOI:** 10.3390/molecules31081268

**Published:** 2026-04-12

**Authors:** Umair Sayad, Shafiq Ur Rahman, Atif Ali Khan Khalil, Abid Ullah, Shafi Ullah, Sultan Mehtap Büyüker

**Affiliations:** 1 Department of Pharmacy, Shaheed Benazir Bhutto University, Sheringal 18050, Pakistan; umairsayad91@gmail.com (U.S.); shafiq@sbbu.edu.pk (S.U.R.); abid@sbbu.edu.pk (A.U.); 2 Department of Biotechnology, Yeungnam University, Gyeongsan 38541, Republic of Korea; 3 Department of Pharmaceutical Sciences, Faculty of Pharmacy, Superior University, Lahore 53700, Pakistan; shafi.ullah@superior.edu.pk; 4 Department of Pharmaceutical Toxicology, School of Pharmacy, Medipol University, Istanbul 34810, Turkey; sultan.buyuker@medipol.edu.tr

**Keywords:** nanorobotics, nanotechnology, multidrug-resistance (MDR), antimicrobial resistance (AMR)

## Abstract

The efficacy of traditional antimicrobial treatments has been largely compromised due to the high occurrence of multidrug-resistant (MDR) pathogens, therefore underlining the limitations of existing drug delivery mechanisms. Pathogens resist pharmacological treatment via different mechanisms, including efflux pump overexpression, biofilm formation, and enzymatic destruction. The application of nanorobotics or controllable nanoscale devices has gained considerable attention for overcoming shortcomings while connecting biomedical engineering, materials science, and microbiology. Despite advancements in nanomedicine, there is still no suitable nanorobotic system applicable against MDR pathogens. Previous studies highlighted device categories and materials but did not explain the detailed nanorobotic mobility, sensing, and programmability to counteract biological resistance. This review combines cross-disciplinary discoveries to design a mechanistic and translational model for nanorobotics effective in controlling infectious diseases while focusing on the advancements in nanorobotic technologies over the past six years (2020–2025), with emphasis on translational readiness, biosafety issues, scalability, regulation, and their mechanistic ability to overwhelm MDR complications. Databases from different publishers, including PubMed, Scopus, and Web of Science, were used to select studies focusing on the potential of emerging nanorobotic therapeutic technologies, such as magnetic microrobots, catalytic nanoswimmers, and DNA origami nanodevices, and their application to bacterial biofilms and antibiotic drug delivery. Evidence from the literature shows that magnetically driven microrobots, catalytic nanoswimmers, and DNA origami structures can actively destroy biofilms, enhance antibiotic penetration, and perform site-specific antimicrobial administration. Nevertheless, most of these innovations remain in the preclinical or prototype stage, hindered by biosafety issues, immunological reactivity, poor routing precision, energy source optimization, and a lack of regulatory and ethical frameworks, which are major challenges for clinical translation.

## 1. Introduction

Multidrug resistance (MDR)-related bacterial diseases are the most important public health issues of the 21st century. According to the annual report of the World Health Organization (WHO), it is estimated that by 2050, the mortality due to antibiotic resistance will reach up to 10 million, overtaking cancer as the major cause of death [1,2]. The effectiveness of traditional antibiotics is compromised due to the adaptability of microorganisms. Bacteria take advantage of mechanisms such as target change, biofilm formation, enzymatic degradation, and efflux pump activation; this compromises the effectiveness of traditional antibiotics despite nonstop pharmaceutical innovations [3,4]. Because of these evolved defense mechanisms in bacterial cells, standard pharmacological therapies are becoming less effective, especially when handling chronic and biofilm-associated conditions [5].

The traditional medicine system has been largely replaced by alternative nanosystems through innovations in stability, absorption, solubility, and passive targeting mechanisms of micelles, liposomes, and nanoparticles [6,7]. However, these systems still depend on diffusion and are unable to effectively overcome the structural and biochemical barriers related to resistant ailments [8]. Bacterial biofilms are dense extracellular matrices containing polysaccharides, nucleic acids, lipids, and proteins that decrease the absorption of antibiotics, decrease the metabolic activity of embedded bacteria, and generate microenvironments that guard infectious agents from the host defense system [9,10]. Accordingly, a significant therapeutic inconsistency persists between the functional limitations of traditional drug delivery systems and the complex physiological barriers inherent to the infection microenvironment [11]. Nanorobots, in comparison to passive nanoparticles, are highly programmable, externally controllable, and dynamic, enabling them to interact with and target biological tissues in a specific way by self-propulsion and navigation mechanisms [12,13]. These nanorobots accomplish active localization, selective drug release, and microenvironmental sensing of biofilms with the combined help of nanotechnology, bioengineering, and materials science. Their active propulsion ability or reaction to external stimuli, like chemical, auditory, or magnetic fields, permits unprecedented spatiotemporal control over drug delivery, pathogen eradication, and biofilm disruption [14,15].

Despite encouraging experimental results, research in this field remains divided across different disciplines. Device engineering and distinct biomedical uses account for the majority of published research, with limited correspondence across these areas [16]. Still, there are limitations to the transition from the conceptual framework to clinical implementation because of several significant barriers in their large-scale production, immunogenicity, biocompatibility, and energy sources [17,18]. Furthermore, their use in humans is limited because of the lack of ethical accuracy and uniform legal frameworks [19].

From the perspective of multidrug-resistant infections, this review critically examines the transdisciplinary interface between nanorobotic design and biological translation, where the existing literature highlights the need for clinical readiness. The preclinical efficiencies of nanorobotic systems in target-specific antibacterial drug delivery and disruption of biofilms are critically evaluated by synthesizing evidence on the mechanisms, actuation strategies, and materials that underpin these systems and categorizing the translational and regulatory bottlenecks that restrict clinical applications, all based on research work published between 2020 and 2025. In conclusion, research suggests a strategic roadmap that combines adaptive navigation, artificial intelligence (AI), and standardized regulatory procedures to lead the development of clinically viable nanorobotic therapies for infection treatment [20,21,22].

The objective of this review is to provide a critical synthesis and comprehensive analysis of recent developments in nanotechnology and their potential implementation against multidrug-resistant (MDR) microbial infections. In contrast to previous studies that mainly focus on material synthesis or isolated biomedical applications [15,16,17], this work adopts a transdisciplinary approach. By combining principles of microbiology, materials science, and regulatory science, this study designs the evolving interface between nanorobotic design and clinical therapeutics. We propose a design–mechanism–translation framework for navigating the complex landscape, which acts as the structural foundation of this review, guiding the synthesis of the existing literature toward a more cohesive understanding of clinical readiness.

## 2. Scope of This Review

This review provides an analysis of studies on the design, functionalization, biomedical applications and manufacturing processes of nanorobotic systems, along with biofilm disruption and antimicrobial characteristics, that were published between the years 2020 and 2025. Particularly, this review focused on:

(1) Microrobots that are driven magnetically and proposed for biofilm disruption or targeted drug delivery.
(2) DNA origami nanorobotics and biohybrid nanotechnologies, specifically programmed for enzyme or drug delivery.
(3) Nanoswimmers that are designed with catalytic or chemical propulsion and applied in bacterial eradication and infection models.
(4) Hybrid nanocarrier systems that incorporate photothermal, photodynamic or sonodynamic antimicrobial processes.

Research studies that only focused on non-autonomous non-biomedical robotic systems, non-autonomous nanoparticles, targeted anti-cancer nanomedicine or non-infectious therapeutic areas were excluded from this review [11].

## 3. Literature Search, Data Extraction and Synthesis

Both conceptual and experimental data were documented with the help of a narrative review methodology. Systematic literature searches were carried out with the help of the Google search engine, including Google Scholar, Web of Science, PubMed, Science Direct, and Scopus, with manual screening of reference lists from essential publications. Different logical operations and keywords were used to search the literature (microrobots, nanoswimmers, nanorobots, magnetic microbots, DNA origami, biohybrid robots, nanotechnology and antimicrobial, biofilm, resistance, infection, drug delivery and target sites).

This review includes only articles published in English that contain original experimental results, translational debates or design frameworks. Reviews, conference abstracts, and non-peer-reviewed publications were excluded unless they presented novel conceptual or regulatory insights [10,22,23].

The collected articles, complete texts, abstracts, and titles were assessed for quality and applicability. Information about the type, structure, propulsion method and mode, target pathogen or model system, therapeutic payload, and outcome measurements of the nanorobot was extracted from the selected literature. The extracted data were synthesized to establish the following thematic categories:

- Advances in design and mechanics (propulsion, control, and functionalization).
- Biomedical uses, such as biofilm penetration, antibiotic delivery, and synergistic therapy.
- Preclinical outcomes (effectiveness, cytotoxicity, and biocompatibility).

Manufacturing, immunogenicity and regulatory requirements are significant translational barriers. Instead of performing a meta-analysis, this study utilized a qualitative narrative strategy to compare different material systems and treatment approaches. This methodology aligns with established frameworks currently used in technology-driven biomedical reviews, which mainly emphasize mapping research trajectories; rather than quantifying aggregated outcomes, it mainly focuses on the identification of translational gaps [20,24,25].

## 4. Nanorobotic Design—Methodological Rationale

This research methodology integrates studies from biology and engineering to explain how advances occur in nanorobot design and their implementation in clinical practices.

Additionally, this approach helps to identify significant challenges, such as immunological responses, biocompatibilities and regulatory aspects, that are often overlooked in technical research studies [26,27].

In the upcoming era, this methodology will demonstrate that the current research represents more than a mere compilation of available data; it clearly outlines what technical and scientific steps are needed to facilitate nanorobotic antimicrobial systems from laboratory experiments to real medical applications. This includes addressing key obstacles, such as scalability, biocompatibility, regulatory compliance, and clinical validation, to ensure successful integration into future therapeutic strategies.

**Figure 1:**
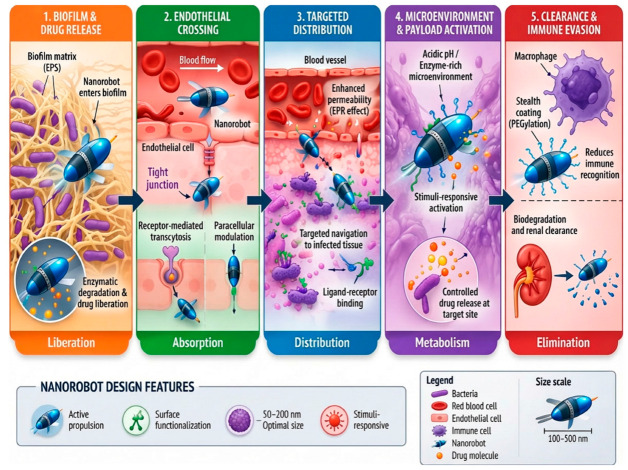
LADME-based design–mechanism–translation framework for antimicrobial nanorobots in MDR therapy. (1) Biofilm and drug release: chemical or mechanical penetration of the extracellular polymeric substance matrix. (2) Endothelial crossing: extravasation via active and passive processes of nanorobots across vascular barriers. (3) Targeted distribution: systemic transportation and localized accumulation of nanocarriers at the infection site. (4) Microenvironment and payload activation: release of antimicrobial agents (e.g., pH-responsive or enzyme-activated) within the specific biochemical conditions of the infection. (5) Clearance and immune evasion: approaches utilized to bypass opsonization and renal/hepatic clearance.

The design of nanorobots for biomedical uses can additionally be rationalized by integrating classical pharmacokinetic parameters, i.e., LADME (Liberation, Absorption, Distribution, Metabolism, and Excretion), which is mostly used in conventional drug discoveries [28]. Regardless of their advanced and active nature, nanorobots still meet these basic biological principles to attain clinical success. The process of drug liberation in nanorobots is achieved by a controlled and stimuli-dependent responsive drug release process, permitting localized therapeutic action [29]. Absorption is improved through active targeting mechanisms and the ability of nanorobots to penetrate deep into physiological barriers, such as cellular membranes and biofilms [30]. The distribution of nanorobots can be governed by their physiochemical characteristics and is affected by directed propulsion systems, such as enzymatic, magnetic or acoustic control, which permit accurate navigation to infection sites [31]. Metabolism is mainly influenced by material properties and composition and addressed through the application of biodegradable and bio-responsive moieties that undergo controlled degradation within biological environments, minimizing cytotoxicity and improving safety profiles [32]. Lastly, excretion of nanorobots depends on size, surface properties, composition and biodegradability, with properly designed nanostructures facilitating clearance through renal and hepatic routes and minimizing long-term accumulation and associated toxicities [33]. This LADME-guided methodology offers a systematic background for aligning nanorobotic design with conventional pharmacokinetic principles, thus improving their translational potential in antimicrobial therapy. The incorporation of LADME principles into nanorobotic design is shown in Figure 1. As shown, each pharmacokinetic module is associated with specific functional landscapes of nanorobots to improve therapeutic efficacy and translational potential.

## 5. Design Principles and Mechanistic Innovations in Nanorobotics

**Figure 2:**
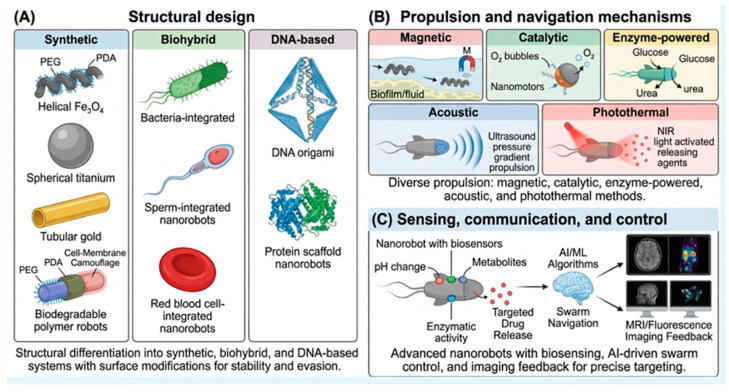
Hierarchical framework for nanorobotics strategies for combating bacterial infections and biofilms. (**A**) Structural design: showing the fabrication of synthetic (polymeric/metallic), biohybrid (cell-integrated), and DNA-based designs tailored for biocompatibility. (**B**) Propulsion and navigation mechanisms: specifying the principles of physical and chemical motion, comprising magnetic field gradients, catalytic fuel conversion, and enzyme-based bio-propulsion. (**C**) Sensing, communication, and control: demonstrating the joined intelligence essential for autonomous navigation, quorum-sensing detection, and external feedback loops.

The development of biomedical nanorobotics resulted from the convergence of bioengineering, nanofabrication and material science. These nanorobots can execute very complex pharmacological tasks at cellular and subcellular levels either autonomously or under external supervision. These nanorobots can easily penetrate physiological membranes and biofilms and deliver their antibacterial content/drug with high precision and accuracy compared to conventional antibiotics [15,34,35]. Diagrammatic presentations of nanorobotics strategies are shown in Figure 2.

## 6. Structural and Functional Design Principles

The biological objective, medicinal payload, and desired propulsion technique of nanorobots largely determine their architectures. Nanorobots can be classified into three primary categories: synthetic, biohybrid, and biological systems [12,18].

Inorganic or polymeric materials, including magnetic iron oxide (Fe_3_O_4_), titanium (Ti), gold (Au), and naturally degradable polymers, are used to create synthetic nanorobots. Nanorobots are usually helical, spherical or tubular in shape, and their movement can be easily influenced and regulated by biological fluids [36]. In contrast, biohybrid nanorobots are composed of both biological and synthetic components, for example, red blood cells, sperm cells, or bacteria, and for locomotion, they use their innate mobility and resemblance with other living microorganisms [37]. Biohybrid nanorobots, which are usually based on DNA nanotechnology, utilize DNA origami frameworks or protein-based skeletons that self-assemble and respond directly to molecular triggers [38,39,40].

Subsequently, biocompatibility influences how effectively an architecture evades the immune system, how long it remains inside the body, and how safely it is used for treatment; therefore, it is crucial for all structures. Various polymers are used as surface coating materials, for example, polyethylene glycol (PEG), polydopamine (PDA), and cell membrane camouflaging, which are used to mask different items and make them more stable and less likely to trigger the immune system of the body [40]. In therapeutic settings, nanorobots can now be improved because of the addition of smart functional domains for drug loading, enzymatic degradation through programmed assembly processes and pH-responsive release mechanisms. Detailed structural and functional aspects of nanorobots are mentioned in Table 1.

## 7. Propulsion and Navigation Mechanisms

Active nanorobots and passive nanocarriers are mechanistically well differentiated from each other by their modes of locomotion and propulsion. Various techniques are employed on micro- and nanoscales for propulsion, including acoustic, chemical, magnetic, catalytic and light-driven systems [46,47].

Since magnetically propelled nanorobots have minimal cellular toxicity, deep tissue penetration, and remote-control capabilities, they are specifically used in biomedical fields. Nanorobots are usually made up of supermagnetic or ferromagnetic materials, which convert oscillating or rotating magnetic fields into translational motion [48]. Helical microbots, which are designed after bacterial flagella, can easily swim through dense fluids and spin, allowing for controlled passage across mucosal barriers and biofilms [49]. Recent studies suggest that magnetic nanorobots carrying antibiotics or enzymes can simultaneously deliver therapeutic drugs and disrupt biofilm materials [50].

**Figure 3:**
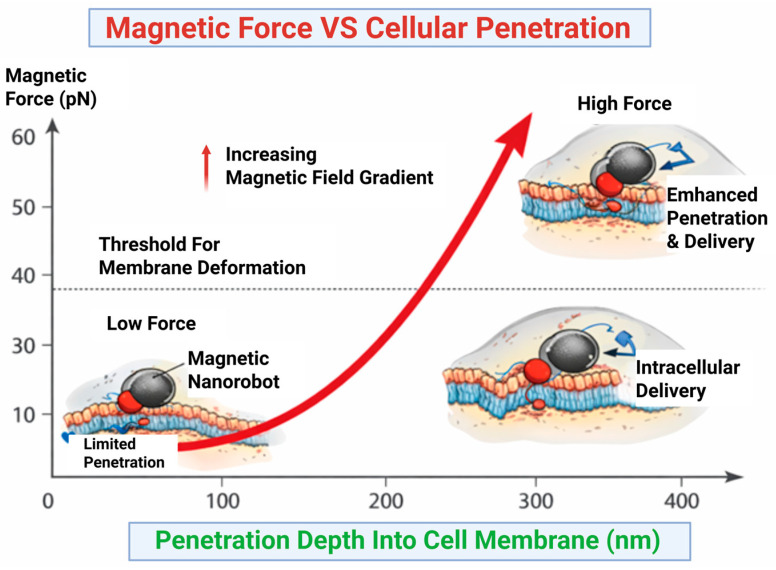
Magnetic force and cellular membrane penetration in magnetically driven nanorobots. Increasing magnetic field gradients increases force generation, enabling membrane deformation and improved penetration depth. A threshold force is required to overcome membrane resistance and achieve intracellular delivery. This highlights the importance of optimizing magnetic parameters for effective nanorobotic transport.

The effectiveness of magnetically propelled nanorobots is governed by the magnitude of the external magnetic force produced under an applied magnetic field. This force (F) can be estimated as directly proportional to the product of volume, magnetic susceptibility and gradient of the magnetic field (∇B), such that F ∝ Vχ∇B. Notably, it is the magnetic field gradient, rather than the field strength alone, that governs the capacity of nanocarriers to produce sufficient force for propulsion and tissue penetration [51]. Quantitative studies have confirmed that increasing field gradients boosts propulsion efficacy and allows nanocarriers to apply mechanical forces capable of altering cellular membranes, thus facilitating uptake and intracellular delivery [52]. It has been reported that optimized magnetic field conditions significantly enhance transmembrane transport efficacy, emphasizing the significance of correlating physical force generation with biological barriers. However, achieving satisfactorily high magnetic gradients in deep tissues remains a major challenge, predominantly under clinically acceptable conditions. Hence, the translation of magnetic nanorobotic systems needs careful optimization of field factors to balance effective force generation with safety and feasibility constraints [51,53]. Figure 3 illustrates the relationship between magnetic force and cellular membrane permeation in magnetically driven nanorobotic systems.

Catalytic and chemical nanorobots utilize chemical energy for propulsion, and with the help of redox reactions, this chemical energy is converted into mechanical work. On the surface of a catalyst, e.g., platinum (Pt) or manganese dioxide (MnO_2_), the hydrogen peroxide (H_2_O_2_) molecule decomposes and creates oxygen bubbles, which help nanorobots move forward [46]. Even though this system can attain high propulsion speeds, its reliance on toxic chemical propellants limits its clinical translation for in vivo application. In recent bioinspired techniques, enzymes are powered by biocompatible fuels like glucose or urea, achieving constant propulsion in physiological settings and thereby overcoming the limitations of cytotoxicity [54].

Acoustic nanorobots utilize ultrasound energy to generate localized pressure gradients, enabling efficient movement through biological tissues with minimal energy dissipation. This method improves translational compatibility by facilitating simultaneous imaging and propulsion of nanorobots using clinical ultra-sonographic devices [55].

Light-driven propulsion based on photothermal or photochemical conversion makes it possible to control space very precisely in clear areas. Gold nanorods and semiconducting nanoparticles absorb near-infrared (NIR) light, which creates localized heat gradients that either move the nanorobot or release antimicrobials [56]. On the other hand, optical scattering in biological tissue makes it hard for them to work in deep tissue.

## 8. Comparative Analysis of Nanorobotic Systems for Multidrug Resistance Infections

**Figure 4:**
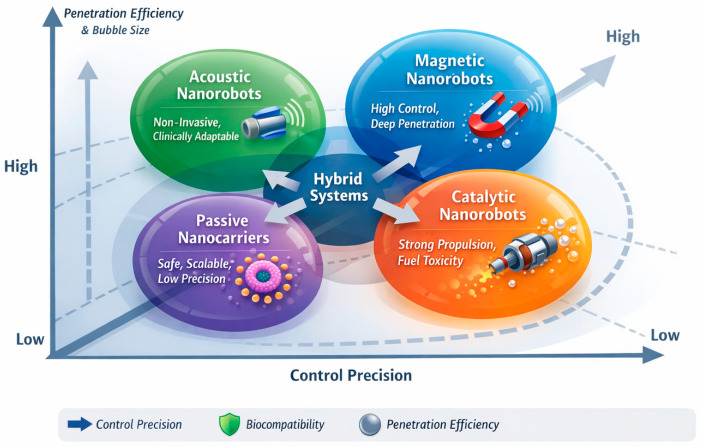
Comparative functional landscape for nanorobot systems against MDR infections. The schematic illustrates four key classes, including acoustic nanorobots (that are non-invasive and clinically versatile with moderate-to-high penetration efficiency), magnetic nanorobots (that can be highly controlled and have high penetration efficiency into tissues), catalytic nanorobots (that are highly propulsive and that may be toxic to the body), and passive nanocarriers (that are highly biocompatible). The axes show the level of control (horizontal) and penetration efficiency (vertical) based on bubble size, indicating trade-offs between various nanorobotic methods.

To clearly contextualize the therapeutic potential of nanorobotics in combating MDR infections, a comparative analysis of various propulsion mechanisms and delivery approaches is essential. The functional variations among major nanorobotic systems are summarized in Table 2 and Table 3 and Figure 4, which highlight trade-offs between control accuracy, biocompatibility and penetration potential.

Magnetically operated nanorobots provide superior external controllability and enhance tissue penetration, making them feasible for targeted drug delivery in cases of deep lesions and infections. However, their reliance on magnetic field gradients and specific equipment restricts scalability and clinical translation [18,51]. On the other hand, catalytic nanorobots have autonomous propulsion and high biofilm penetration capacity; however, they are limited by toxicity concerns linked with chemical fuels like H_2_O_2_ [57,58].

Acoustic nanorobots offer a non-invasive alternative with high biocompatibility and clinical compliance due to the extensive applications of ultrasound technologies. Nevertheless, their relatively minimum propulsion limit and force spatial accuracy may restrict their effectiveness in dense biofilm milieu [59]. Passive nanocarriers, which are highly safe and scalable, lack active targeting capacity and exhibit low penetration efficiency, especially in complex infection sites [60,61].

A critical view from this perspective shows that no single nanorobotic system is universally optimal; rather, therapeutic potential depends on aligning a system’s framework with a specific pathological condition, such as biofilm density, infection site and required penetration ability. This highlights the significance of evolving hybrid systems that integrate different propulsion mechanisms to overcome individual limitations and increase overall therapeutic potentials.

The comparative landscape of nanorobots against MDR infections demonstrates that magnetic nanorobots show high accuracy and great tissue penetration, yet they are constrained by external magnetic fields. Although catalytic nanosystems have high propulsion and biofilm penetration, they have limited biocompatibility because of their reliance on chemical fuels. Acoustic systems provide a non-invasive and biocompatible substitute with moderate propulsion efficiency, while passive nanocarriers demonstrate high biocompatibility and scalability but lack active targeting and penetration abilities.

## 9. Sensing, Communication, and Control

Functional intelligence, or perceiving and reacting to environmental signals, is a key component of next-generation nanorobotics in addition to mobility. Modification of nanorobots with biosensors results in the real-time detection of various biomarkers, such as bacterial metabolites, enzyme activity and pH, causing changes in various behaviors like alteration in locomotion or release of drug substances [62].

Machine learning methodologies and artificial intelligence-based control algorithms have distinctly advanced the predictable navigation of nanorobotic swarms inside highly complex biological microenvironments [63]. These computational algorithms lead to the optimization of navigational trajectories, suppressing nonspecific target interactions and enhancing therapeutic thresholds through a real-time feedback mechanism caused by imaging modalities like magnetic resonance imaging (MRI), fluorescence microscopy and tomography.

## 10. Mechanistic Innovations for Antimicrobial Therapy

**Figure 5:**
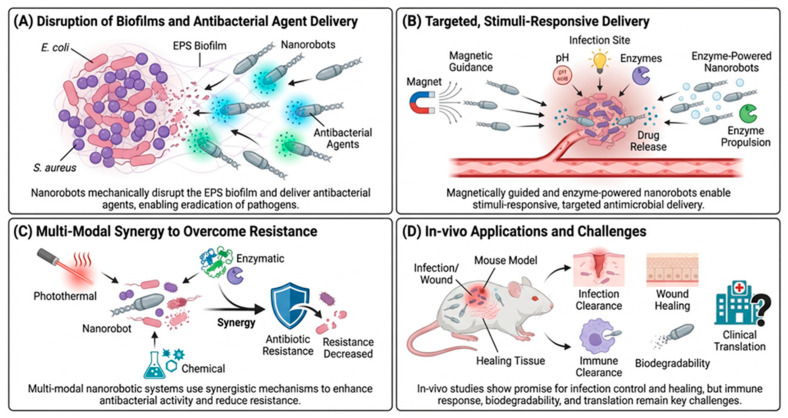
Nanorobot-enabled strategies for biofilm disruption, targeted antimicrobial delivery, and infection control. (**A**) Penetration of the extracellular polymeric substance matrix and delivery of the antimicrobial agent to a localized site. (**B**) Activation of payloads in response to different endogenous or exogenous stimuli. (**C**) Integrated effect of mechanical disruption and synergistic drug combinations to bypass bacterial efflux pumps. (**D**) Transition from laboratory models to systemic circulation.

The utilization of proposed principles for the control of infections signifies one of the most critical innovations in the field of biomedical nanorobotics. To overcome multidrug resistance (MDR)-related challenges, nanorobots utilize three principal strategies, as shown in Figure 5.

1. Mechanical biofilm disruption: Bacteria are exposed to antimicrobial chemicals, and biofilm matrices are broken up by rotational or oscillatory motion [64].
2. Optimization of local drug delivery: Systemic exposure is reduced by permitting the direct delivery of enzymes or antibiotics to infected microenvironments [22].
3. Multimodal synergistic effect: Combining physical disruption with chemical or photothermal bactericidal actions can increase killing power even against populations of bacteria that are latent [19].

Magnetically driven Fe_3_O_4_-PDA nanorobots and DNA origami constructs equipped with lysozyme or antimicrobial peptides have the potential to remove up to 90–95% of biofilm mass in vitro when compared to traditional nanoparticle therapies [16].

## 11. Overcoming Physiological Barriers

Overcoming various physiological barriers is the key challenge for nanomedicine, as such obstacles significantly impede the efficient delivery of therapeutic agents to a targeted site [65]. Within the human body, nanorobots must navigate diverse biological interfaces, such as endothelial layers of vessels, cell membranes and organ-specific barriers, that strongly regulate molecular transport [66]. Nanorobotic systems have unique advantages in addressing challenges because of their active propulsion mechanisms and tailorable physicochemical characteristics. For instance, externally directed nanorobotic systems, like magnetic, enzymatic or acoustic fields, can generate localized mechanical forces that increase their penetration through endothelial membranes [67]. Furthermore, surface functionalization of nanorobots with special ligands allows for receptor-mediated endocytosis, facilitating targeted cellular uptake and enhancing tissue localization [68].

**Figure 6:**
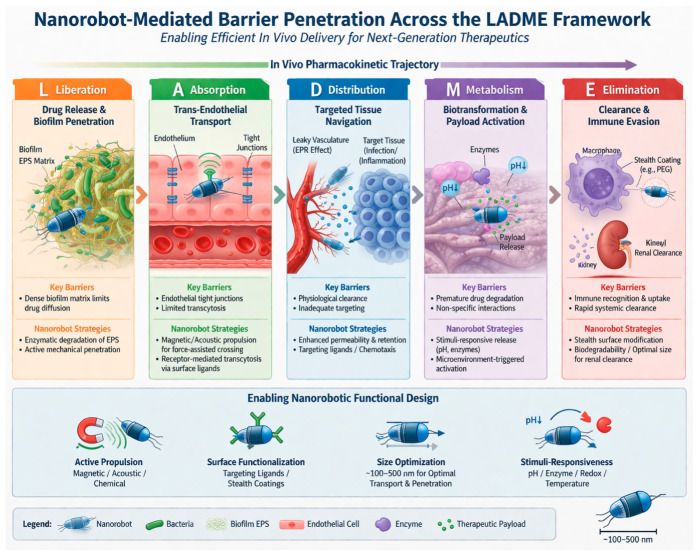
Schematic representation of nanorobot-mediated penetration through physiological barriers across sequential stages, such as biofilm disruption, endothelial crossing, targeted tissue distribution, and stimuli-responsive payload activation.

Additionally, their nanoscale dimensions and specially engineered surface characteristics enable them to exploit properties such as enhanced permeability and retention effects, particularly in inflamed or infected regions [69]. From the perspective of MDR infections, these systems have shown the ability to infiltrate dense biofilm structures, which are otherwise very resistant to conventional antimicrobial therapies [70]. Regardless of all these promising capacities, challenges exist, such as immune system recognition, complexity of in vivo navigation and clearance problems; thus, overcoming physiological barriers requires an integrated landscape combining mechanical propulsion, adaptive material engineering, and biochemical targeting to achieve maximum therapeutic efficacy [71]. Figure 6 illustrates how nanorobots navigate key physiological barriers through synchronized transport and targeting mechanisms, thereby highlighting the potential to improve delivery efficiency in vivo.

## 12. Design-to-Translation Considerations

Despite the quick advances in mechanics, nanorobots must be manufactured, scaled, and biocompatible for their transition from lab prototypes to clinical treatments. Additive manufacturing and 3D microprinting have made it possible to build microhelices and tubular robots repeatedly with nanoscale precision [72]. Concurrent research is being done on hydrogel–metal composites and other hybrid materials for biodegradable designs that eliminate the potential for long-term retention in vivo [73].

Biocompatibility remains a critical, yet evolving aspect of nanorobotic systems. Current advances in surface modification, including coating with polymers, utilization of biodegradable structural moieties and ligand conjugation, have enhanced biological compatibility while reducing cytotoxicity [60]. These changes increase circulation stability and decrease nonspecific interactions with healthy tissues. Despite these modifications, several challenges remain. Nanorobots may still evoke immune system responses, causing rapid clearance or unintended inflammatory reactions [60]. Furthermore, concerns related to long-term accumulation, potential cytotoxicity, and biodegradation of certain moieties, particularly in catalytic systems, remain significant barriers to clinical transition. Hence, biocompatibility should be considered a topic of ongoing progress rather than a fully resolved issue, demanding further evaluation through standardized preclinical and clinical investigations [61,74].

Nevertheless, there are still limitations like standardizing testing procedures, immunological tolerance, and ensuring steady propulsion under physiological conditions. In order to create nanorobots and consider these problems, a comprehensive framework that integrates computational design, regulatory harmonization, and advanced material innovation is needed [75].

## 13. Translational Roadmap for Nanorobotic Systems in MDR Infections

**Figure 7:**
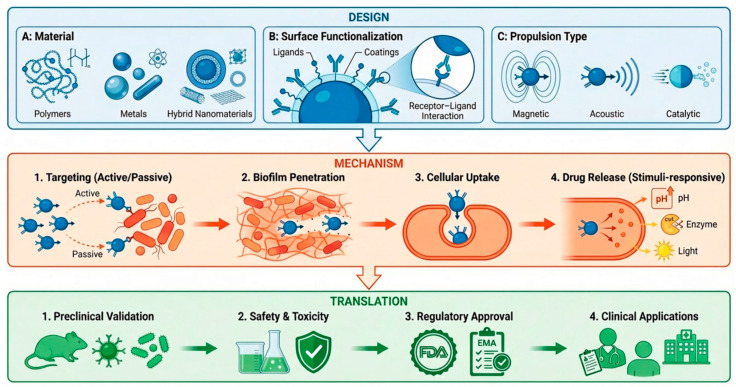
Nanorobot design–mechanism–translation framework for combating MDR infections. The nanorobotic design framework includes material selections such as polymers, hybrid systems and metals, surface functionalization, like coating and ligands, and propulsion mechanisms, like acoustic, catalytic and magnetic. These parameters regulate mechanistic behaviors such as active or passive targeting, biofilm eradication, cellular penetration and stimuli-responsive drug release triggered by environmental stimuli like enzymes, pH, or external fields. The transition stage includes preclinical validation, safety and toxicity assessments, regulatory approval and clinical applications.

The evolution of nanorobots from laboratory innovation to clinical utilization (Figure 7) requires a structured elucidation across preclinical studies, regulatory feasibility and realistic clinical approaches. To increase the translational significance of this review, key elements, including preclinical benchmarks, regulatory pathways, and clinical entry points, are critically considered.

At the preclinical benchmark phase, four critical sites are highlighted. The first one is targeting efficiency, including accurate localization and incapacitating physiological barriers, mostly achieved through externally guided nanorobotic systems [58]. The second is drug loading and release, emphasizing a stimuli-responsive system with precise release kinetics for targeted therapy [76]. The third is toxicity thresholds, pointing out cytotoxicity and immunogenicity to ensure biosafety and biocompatibility [77]. The fourth one is biofilm penetration and antimicrobial effect, suggesting that nanorobots can easily penetrate and eradicate biofilms, which serves as a key benchmark distinguishing them from conventional therapies [78].

The clinical translation of nanorobotic systems is further evaluated by regulatory authorities such as the FDA/EMA. These regulatory framework considerations outline important translational requirements, such as combination product classification integrating device and drug functionality challenges [79], safety and toxicity profiling held by long-term validation studies [80,81], and clinical trial design, emphasizing end points like infection elimination and biofilm eradication [82].

Among the current technological limitations, the abrupt application of nanorobotic systems to combat systemic infections remains challenging. Therefore, identifying feasible initial clinical targets is crucial. At the clinical level, the therapeutic landscape encompasses several potential domains, such as localized infections, biofilm-associated diseases requiring targeting disruption and topical drug delivery approaches aimed at limiting systemic toxicity [58,83].

## 14. Biomedical Applications of Nanorobotics in Multidrug-Resistant Infections

Traditional antimicrobial therapy is limited by microorganisms through different resistance mechanisms, like biofilm formation, phenotypic persistence, horizontal gene transfer and hiding drug-binding sites, which explains the clinical burden of multidrug-resistant infections (MDRIs) [84,85]. Nanorobotic systems represent an emerging therapeutic potential transition from conventional passive nanocarriers to adaptive, intelligent nanotherapeutic platforms because they can actively navigate biological settings, mechanically break biofilm formations, and perform on-demand antimicrobial administration [35]. This section critically examines the preclinical and experimental applications of nanorobots in the primary models of infectious diseases, as well as their relative advantages, translational challenges, and mechanistic effectiveness.

### 14.1. Disruption of Bacterial Biofilms

Highly organized bacterial populations known as biofilms are protected from host immunological reactions and antibiotic activity by an extracellular polymeric matrix that the bacteria manufacture on their own. More than 80% of chronic infections, such as wound infections, pneumonia, and device-associated sepsis, are linked to biofilms [86]. The inability of conventional antibiotic treatments to adequately permeate this matrix commonly leads to prolonged infections and partial bacterial clearance.

Nanorobotic systems are remarkable because they can distribute antibacterial chemicals and physically clear biofilms. For instance, magnetically driven microrobots can increase the dispersion and effectiveness of antibiotics by employing oscillating magnetic fields to bore through biofilm layers [36]. Fe_3_O_4_-polydopamine nanorobots coated with tannic acid outperformed passive nanoparticles in reducing biofilm biomass against *Escherichia coli* and *Staphylococcus aureus* by 95% [73].

Similarly, biofilm eradication has been programmed using DNA origami nanorobots. DNA nanocages have been engineered to be activated by bacterial quorum-sensing molecules, which contain lysozyme, an enzyme capable of degrading bacterial cell walls. Eighty to ninety percent of MRSA biofilms and multidrug-resistant *E. coli* were successfully destroyed by these nanorobots [87]. These structures’ adaptability mitigates off-target effects, enabling precision-targeted recognition and payload release within the infected microenvironment.

### 14.2. Targeted Antimicrobial Delivery

Conventional nanocarriers passively aggregate at the site of infection by relying on the increased permeability and retention (EPR) effect, which is sometimes unreliable in tissues with little vascularization or variety [6]. However, therapeutic medications can be delivered directly to infected microenvironments using nanorobots, which can travel under external magnetic or acoustic fields with spatiotemporal accuracy. Under various field strengths, magnetic nanorobots have demonstrated controlled release of antimicrobials. For example, hydrogel microrobots with antibiotic payloads were developed, which released their payloads upon encountering pH gradients typical of pathological tissues. These nanorobots also reduced biofilm regrowth in *S. epidermidis* infection models by 90% [88].

Another development is enzyme-powered nanomotors, which use endogenous substrates such as glucose or urea for both propulsion and concurrent therapeutic activation [89]. In infected wound models, catalase-driven nanomotors functionalized with ciprofloxacin were used to rapidly destroy bacteria within 6 h compared to 24 h for free drug therapy [90].

Moreover, hybrid nanorobots offer double advantages of chemical self-propulsion and directional control. They combine catalytic propulsion and magnetic navigation, allowing them to penetrate deeper into biofilms and tissue layers [62,91]. These systems are used in vitro to attack *Pseudomonas aeruginosa* biofilms, practically performing a complete breakdown and showing bactericidal effects [73].

### 14.3. Combination and Synergistic Therapies

Recent studies demonstrate that the most potent antibacterial effects come from multimodal approaches that combine photothermal, chemical, and mechanical mechanisms. For example, light-driven gold nanorobots, when mixed in bacterial cultures, caused localized hyperthermia upon exposure to near-IR radiation and, at the same time, they released antibiotics to completely accomplish biofilm eradication [56].

Similarly, enzyme-loaded catalytic microrobots that co-deliver DNase and antibiotics enhance bacterial sensitivity and work in concert to degrade the extracellular polymeric substance (EPS) matrix. In polymicrobial models, the method produced a 70% decrease in total biofilm biomass [92].

The risk of future resistance development is reduced by enhancing bactericidal effects and lowering antibiotic doses through these synergistic mechanisms. Notably, multi-model nanorobots are engineered to respond to environmental stimuli such as bacterial enzymes or oxidative stresses, enabling site-specific activation [93].

### 14.4. In Vivo and Translational Models

The use of nanorobots in in vivo environments is still rare but rapidly evolving despite widespread in vitro success. Current studies on animal models have shown encouraging results in terms of infection clearance, wound healing and improved survival. Similarly, it has been reported that magnetically guided nanorobots decrease bacterial burden and inflammation in animal models of chronic wound infection without causing any marked injury.

Biohybrid nanorobots have shown great potential for stimulating natural propulsion by utilizing living cells or microorganisms as their propulsion energy. It has been reported that bacteria-driven microrobots with antibiotic nanoparticles may independently target hypoxic infection sites, utilizing bacterial chemotaxis to navigate across complex tissue environments [37].

The transition from preclinical to clinical application remains hindered by several critical challenges, including immunological clearance, biodegradability, and long-term biodistribution [66,94]. Furthermore, the absence of standardized infection models for evaluating nanorobotic efficacy limits the comparability of results across different studies [95]. To address these challenges, interdisciplinary collaboration is essential for the development of standardized benchmark models, ethical frameworks, and Good Laboratory Practice (GLP) protocols specific to nanorobotics.

### 14.5. Comparative Advantages of Nanorobots over Conventional Nanomedicine

Nanorobots have several mechanistic and therapeutic advantages over passive nanocarriers:

- Physical rupture of biofilms, which increases bacterial exposure and antimicrobial penetration.
- Controllable, on-demand medication delivery that enables therapeutic modification in real time.
- The potential for theragnostic (therapy + diagnosis) applications through interaction with diagnostic modalities.
- Overcoming diffusional constraints through active navigation across viscous biofilm matrices.
- Less systemic toxicity because medication release mostly takes place at infection sites [7,25].

For instance, while liposomal and polymeric nanoparticles typically exhibit penetration depths of less than 20 µm, magnetically operated nanorobots can exceed 100 µm, resulting in much higher rates of bacterial reduction in mature biofilms [72]. The comparative analysis in Table 4 reveals that active nanocarriers show active propulsion, bioactive-triggered drug release, biofilm disruption and navigation, while their biosafety requires further evaluation and has limited manufacturing scalability. On the other hand, conventional nanocarriers have passive mobility, well-established biosafety protocols, diffusion and pH-triggered drug release, and high manufacturing scalability but limited biofilm penetration. These results suggest that nanorobotics could revolutionize the therapeutic management of chronic infections by replacing empirical antibiotic treatments with highly customized, precision-based therapies.

### 14.6. Future Prospects and Current Limitations

Although nanorobotics represents a revolutionary advancement in infection control, the practical application of this technology remains in its early stages. Large-scale repeatability, in vivo navigation accuracy, the standardization of propulsion mechanisms, and long-term biosafety are currently the primary constraints [26]. Moreover, regulatory challenges pertaining to active nanosystems, which are classified as drugs, devices, and biological products, continue to impede clinical approval [96].

To fully utilize the potential of nanorobots in the fight against multidrug-resistant ailments, future research must focus on a few crucial areas. Currently, the first and most urgent need is the development of biodegradable, nontoxic propulsion mechanisms that offer biocompatibility and safety in clinical settings. Second, to achieve precise navigation and targeted therapeutic action inside complex biological milieus, AI-guided control systems and real-time imaging technologies must be integrated. Third, to validate the reproducibility, efficiency, and long-term safety of nanorobotic interventions, reliable infection and safety models must be established. Finally, in addition to these, the development of hybrid regulatory frameworks is needed to address the unique features of active nanotherapeutics, which usually blur the conventional boundaries between drugs, medical devices, and biological products. As synergistic collaborations among nanotechnology, microbiology and robotics grow, it is anticipated that nanorobots will progress from laboratory prototypes to promising therapeutic strategies under active investigation for precise antimicrobial treatments, leading to a new era in the treatment of multidrug-resistant ailments.

## 15. Translational Barriers and Regulatory Challenges in Clinical Nanorobotics

The clinical implementations of nanorobots remain limited despite substantial preclinical advancements due to a complex array of biological, technological and regulatory complications. None have progressed to either clinical trials or human use despite the fact that many in vitro and in vivo animalstudies have demonstrated robust antimicrobial efficacy. This translational bottleneck highlights the multidisciplinary character of nanorobots, which lies at the nexus of materials science, engineering, and medicine fields that have conventionally worked under different developmental and regulatory models.

### 15.1. Biocompatibility and Biosafety Concerns

Biocompatibility is one of the major challenges in nanorobotics for treating diseases; these challenges arise from complex interactions with various biological systems, including immune system recognition, protein corona formation, and clearance dynamics [97]. Furthermore, nanorobots that contain inorganic materials, such as metals like titanium, iron and nickel, face various problems such as long-term tissue accumulation, cytotoxic ion release and oxidative stress [46]. On the other hand, in various physiological environments, hydrogel- or polymeric-based nanorobots, which are becoming more biocompatible, may break too easily and encounter structural variability [73].

In order to address these challenges, scientists have explored biodegradable hybrid materials that combine metal cores with bio-resorbable polymers or biomimetic coverings such as extracellular vesicles and erythrocyte membranes [98]. Such surface activation can enhance the duration of circulation, increase the targeting capability of infection sites and reduce immune system activation. The current literature still lacks a comprehensive toxicological profile that includes long-term biodistribution, acute and chronic effects, and reproductive toxicity [99].

On the other hand, nanorobots operated by fuel raise extra safety concerns; for example, nanorobots that are propelled by chemicals or utilize reactive fuels, such as hydrogen peroxide, may generate destructive by-products, which make them unsafe for systemic applications [100]. However, switching to magnetically driven nanorobots or enzymatically propelled nanorobots is one of the biggest successes, although evolution towards clinical applications needs strict and more detailed pharmacokinetic and pharmacodynamic investigations [54].

### 15.2. Immunogenicity and Host Interaction

The human defense system poses a significant threat to the functionality and durability of nanorobots. The therapeutic efficacy of these nanorobots may be compromised by their rapid sequestration in the spleen and hepatocytes, following recognition by the reticuloendothelial system [101]. Moreover, hypersensitivity reactions may be triggered by inflammatory cascades, cytokine release, and complement activation [102].

Recent technological innovations adopt cell membrane coating techniques, which wrap nanorobots with a natural cellular membrane, similar to macrophages or platelet membranes, thereby providing immune system escape and pathogen-targeting abilities [103]. These biohybrid advancements bridge the gap between biological and artificial systems, allowing “stealth” nanorobots to function in complex physiological environments; however, this same bio-integration complicates monitoring, classification and long-term biosafety evaluation [104].

### 15.3. Manufacturing and Scalability Constraints

There are numerous manufacturing-related production limitations with nanorobots/Specifically, traditional means of nanofabrication, such as electrochemical synthesis, lithography and template-assisted deposition, are highly laborious, offer low yields and are challenging to scale for clinical-grade production [49]. Additionally, multifunctional nanorobots that incorporate drug loading, sensing, and propulsion processes are gradually becoming more varied and complex.

New technologies bring developments in the accuracy and reproducibility of nanorobot structures and enhanced microfluidic templating, assembly and three-dimensional (3D) nonprinting [25]. For instance, high-throughput 3D printing of magnetic microhelices with near-atomic precision was demonstrated, allowing for the mass production of standardized microrobots for treatment purposes [72]. However, it remains technically and economically challenging to ensure sterility, batch uniformity, and traceability at the nanoscale level; consequently, many of these developments fall short of Good Manufacturing Practice (GMP) standards [105].

The absence of International Organization for Standardization (ISO) compliant testing agendas and established quality-control procedures for active nanosystems is a substantial drawback. Reproducibility and cross-laboratory justification are challenging due to the lack of globally recognized standards, which delays regulatory approval [106].

### 15.4. Regulatory and Ethical Complexities

The regulatory ambiguity surrounding nanorobotic therapy is likely the most significant translational obstacle. There are currently no clear classifications for “active nanomedical devices” that combine mechanical and pharmacological capabilities of regulatory bodies like the European Medicines Agency (EMA) and the Food and Drug Administration (FDA). Therefore, nanorobots often belong to one of three groups: biological products, drug–device combinations, or medical devices [107,108].

### 15.5. Translational Pathway and Clinical Integration

In order to bridge the gap between experimental discovery and clinical implementations, a system-level translational approach is required that takes safety, regulatory compliance, and production aspects into consideration. This process can be facilitated by a suggested three-phase model:

- Preclinical Standardization: Generating dose–response relationship profiles distinctive to active nanosystems, toxicity benchmarks, and authenticated infection models.
- Integrated Regulatory Framework: Working together, academia, industry, and regulatory bodies launch classification standards and approval measures for nanorobotic treatments.
- Clinical Translation: Restricted, biodegradable systems are given importance in phase I/II human clinical trials to assess safety and efficacy in a clear infectious disease model.

Through the synergy of artificial intelligence-driven modeling and digital twin simulations, manufacturers can design nanorobot–host interactions prior to in vivo experiments, thereby improving risk assessment and prognostic performance [25,109].

Meanwhile, cross-sector cooperation involving scientists, physicians, microbiologists, and legislators is essential to establish an environment where nanorobots can progress from experimental prototypes into clinically controlled therapeutic settings [19].

## 16. Future Directions and Strategic Roadmap

Nanorobots have emerged as a prominent field for the treatment of infectious diseases due to the synergy between nanotechnology robotics and biomedical research. To ensure their emergence from conceptual experimental innovation to clinical practice, a careful multi-faced strategy is required that coordinates scientific discovery with technological scalability, ethical oversight and biosafety assurance. Whether nanorobots develop into therapeutically useful antibacterial agents or stay a laboratory curiosity will be determined over the course of the next ten years. Some of the reported disadvantages in specific areas, along with research directions, are represented in Table 5.

### 16.1. Material Innovation and Biodegradable Design

The development of biocompatible and biodegradable nanorobotic platforms that can operate and be cleared safely in vivo is a top strategic priority. Transient therapeutic activity without long-term residue can be achieved by combining bioinspired propulsion systems with naturally generated materials such as silk fibroin, chitosan, and collagen [110]. Environmentally safe and physiologically resorbable nanorobots are being made possible by developments in transient electronics and biodegradable magnetic composites [111].

Newly developed self-assembling nanorobots that are powered by hydrophobic contacts or molecular recognition may further simplify production while guaranteeing exact control over shape and functionality [112]. In the meantime, AI-guided material design platforms are accelerating the prediction of the optimal degradation kinetics, toxicity profiles, and nanorobot–tissue interactions [25].

In order to reduce the trial-and-error cycle in material discovery, future research should focus on closed-loop material optimization, merging computational design, automated synthesis, and real-time biological feedback [113].

### 16.2. Integration of Artificial Intelligence and Real-Time Navigation

The integration of machine learning (ML) and artificial intelligence (AI) into nanorobotic control and navigation systems represents the second pillar of progress. AI may be utilized to optimize propulsion settings, forecast infection site microenvironments, and dynamically modify nanorobot trajectories in response to physiological feedback [114,115].

To simulate nanorobot–host interactions and forecast negative consequences prior to clinical testing, digital twin frameworks—virtual reproductions of biological systems—are becoming increasingly popular [109]. By accelerating the development of secure and effective delivery algorithms, these technologies may improve accuracy while reducing the need for trial and error.

Furthermore, integrating AI-assisted magnetic field programming with real-time imaging modalities (such as photoacoustic tomography and magnetic particle imaging) could enable closed-loop navigation systems, transforming nanorobots into intelligent, self-correcting therapeutic agents [58].

### 16.3. Biohybrid and Immunomodulatory Nanorobots

The development of biohybrid nanorobots offers unparalleled possibilities for adaptive infection control by fusing artificial components with biomimetic structures or living cells. Integrating immune cells, bacteria, or extracellular vesicles as functional components enables self-propulsion, environmental detection, and pathogen-specific targeting [116].

In the future, in addition to administering medications, nanorobots may be able to directly transport immunomodulatory compounds into infection microenvironments or enhance phagocytic clearance to actively alter immune responses [26]. Combining antibacterial administration with immunologically reprogrammable, dual-function nanorobots has the ability to completely cure chronic infections.

However, these systems demand comprehensive monitoring strategies during early development as they raise concerns regarding ethical consent, biosafety, and controllability [96].

### 16.4. Regulatory Harmonization and Risk–Benefit Frameworks

Innovations in global regulatory frameworks must keep pace with technological advancements. The lack of international standards for nanorobots is one of the key problems in their clinical translation [108]. To consider the twin mechanical and pharmacological nature of active nanosystems, risk–benefit development methods specific to these systems should be merged into future frameworks [27].

A proposed Global Nanorobotic Regulatory Alliance (GNRA) might bring together EMA, FDA, WHO, and International Organization for Standardization (ISO) partners to create:

- Differentiation between autonomous and externally operated nanorobots through tiered safety testing procedures.
- Standardized reporting formats for long-term biosafety, toxicity, and clearance.
- Adaptive ethical review procedures that take patient consent, data privacy, and autonomy into account.

When legislation is implemented, open-access registries for clinical data from nanorobotics should be set up to promote transparency, reproducibility, and post-market monitoring [117].

### 16.5. Clinical Translation Pathways and Multidisciplinary Collaboration

Nanorobotics must use successive clinical translation frameworks to get from laboratory research to bedside applications. Research should concentrate on localized infections, such as implant-associated infections, osteomyelitis, and wound biofilms, where systemic risk is reduced through regulated distribution and direct access [117].

To guide this translation, networks of regulatory scientists, physicians, engineers, and microbiologists must collaborate. National or regional Nanorobotic Translational Consortia (NTC) may be formed to provide a shared infrastructure for clinical prototyping, imaging, and safety testing in order to expedite the bench-to-bedside development process [72].

Integration with ongoing antimicrobial stewardship initiatives will ensure that nanorobotic treatments support responsible and long-term therapeutic use by enhancing, rather than replacing traditional antibiotics [7].

### 16.6. Ethical, Environmental, and Societal Dimensions

The ethical and environmental implications of using nanorobots in biomedicine must be considered. Unprecedented concerns are raised by the confinement, retrieval, and ecological impact of self-propelled autonomous nanosystems [58]. “Nanoethics-by-design,” which integrates ethical considerations into early engineering stages, as opposed to post hoc regulation, is a necessary component of bioethical governance [118].

Public approval will be determined by the transparent disclosure of the risk-to-benefit ratio. Technological developments alone will not be sufficient for long-term success; global collaboration, multidisciplinary oversight, and participatory ethics will also be needed to preserve public trust [64].

### 16.7. Vision: The Nanorobotic Therapeutic Ecosystem

In 2035, programmable nanorobots will operate with high accuracy inside the physiological systems of the human body and will be digitally connected, powered by artificial intelligence, and ethically regulated, which forms the conceptual basis for nanorobotic therapies. These systems will connect with other nanomedical systems through biocompatible communication networks to autonomously diagnose, treat, and monitor ailments [25,119].

Conclusively, infection therapies will shift from reactive intervention to proactive, adaptive infection control due to the integration of robotics, molecular medicine and data science.

## 17. Conclusions and Prospects

Nanorobots bring a paradigm revolution to the field of therapeutic monitoring of multidrug-resistant ailments, bridging active biomedical engineering with nanomedicine. In comparison to traditional drug carriers, nanorobots provide higher therapeutic precision due to their tailored antimicrobial delivery, biofilm eradication capabilities, and self-propelling power. However, their therapeutic efficacy is compromised primarily particularly by challenges in biocompatibility, scalable manufacturing, immune system evasion and the limitations of standardized regulatory frameworks. To address these multifaceted issues, it is necessary to integrate research that bridges material innovation, translational biology and computational design.

In the future, manufacturing smart, biodegradable and ethically regulated nanosystems that can safely perform their roles within the human physiological environment will pave the way for developing clinical nanorobots. Achieving this goal will depend mainly on adaptive navigation, artificial intelligence (AI), and the incorporation of biohybrid designs. Nanorobots have the capacity to redefine the limits of existing medicine by transforming infection therapy from a reactive practice into a precise, adaptable, and preventive discipline through multidisciplinary collaboration and coordinated global standards.

Nanorobotics represents a promising strategy under active investigation; however, it is still an evolving approach for combating MDR infections. Although substantial advances have been made in mechanistic and design frameworks, significant challenges persist in scalability, clinical translation, and biocompatibility. Future studies should emphasize standardized evaluation frameworks, interdisciplinary collaboration, and regulatory alliances to facilitate the translation from experimental disciplines to clinically viable therapies.

## Figures and Tables

**Table 1:** Structural and functional design principles of nanorobots.

Nanorobot Type	Propulsion Mechanism	Typical Material	Actuation Control	Biocompatibility	References
Magnetic microbots	External magnetic field	Fe_3_O_4_, NiTi alloy	Remote, precise	Moderate	[41]
Catalytic nanoswimmers	Chemical (H_2_O_2_, urea)	Pt, Au	Autonomous	Low (H_2_O_2_ toxicity)	[42]
Acoustic nanorobots	Ultrasound field	Gold–polymer composites	Remote, deep tissue	High	[43]
DNA origami robots	Programmable folding	DNA nanostructures	Enzyme trigger	Excellent	[44]
Biohybrid robots	Bacteria/cell-driven	Living cell membrane	Chemotactic	Variable	[45]

**Table 2:** Comparative analysis of nanorobotic systems for MDR infections.

Feature	Magnetic Nanorobots	Catalytic Nanorobots	Acoustic Nanorobots	Passive Nanocarriers
Propulsion Mechanism	External magnetic field	Chemical reactions (e.g., H_2_O_2_)	Ultrasound waves	Diffusion/physiological gradients
Control Precision	High (external control)	Moderate (self-propelled)	Moderate	Low
Penetration Ability	High (deep tissue)	High (localized propulsion)	Moderate	Low
Biocompatibility	Good (depends on coating)	Limited (fuel toxicity concerns)	High (non-invasive)	High
Energy Requirement	External	Internal (chemical fuel)	External	None
Clinical Feasibility	Moderate (equipment-dependent)	Low (toxicity issues)	High (clinically adaptable)	Very high
Advantages	Precise targeting, deep penetration	Autonomous movement	Non-invasive, safe	Simple, scalable
Limitations	Equipment constraints	Toxic fuels, instability	Lower force generation	Poor targeting
Best Use Case	Deep infections, targeted therapy	Biofilm disruption	Localized therapy	General drug delivery

**Table 3:** Comparative analysis of nanorobotic systems, their major limitations and uses.

System Type	Propulsion Mechanism	Key Advantages	Major Limitations	Best Clinical Use	References
Magnetic	External magnetic field	High-precision targeting; deep tissue penetration	Requires strong magnetic gradients; equipment dependency	Implant infections; deep tissue targeting	[18,51]
Catalytic	Chemical reactions (e.g., H_2_O_2_)	Autonomous motion; strong biofilm penetration	Fuel toxicity; poor biocompatibility	Biofilm disruption (localized use)	[57,58]
Acoustic	Ultrasound-driven	Non-invasive; clinically adaptable; safe	Lower propulsion force; reduced precision	Localized infections	[59]
Passive	Diffusion-based	High safety; scalable; simple	Poor targeting; limited penetration	Systemic delivery	[60]

**Table 4:** Comparative analysis of nanorobots over conventional nanomedicine and their clinical relevance.

Parameter	Conventional Nanocarriers	Active Nanorobots	Clinical Relevance
Mobility	Passive (Brownian motion, EPR effect)	Active propulsion (magnetic, chemical, acoustic)	Enables targeting deep biofilms/infection sites
Biosafety	Generally established	Requires further evaluation	Toxicity studies ongoing
Regulatory Framework	Existing for nanomedicine	Under development	Expected within next 5–10 years
Drug Release	Diffusion or pH-triggered	On-demand, externally or biomarker-triggered	Controlled spatiotemporal release
Biofilm Penetration	Limited	Mechanical disruption and navigation	Effective against dense MDR biofilms
Manufacturing Scalability	High	Limited, improving with 3D microprinting	Gradually increasing feasibility

**Table 5:** Present drawbacks, research directions and clinical research plan.

Key Areas	Present Drawbacks	Research Direction/Proposed Plan
Biocompatibility	Metallic toxicity	Use of polymeric and biodegradable materials
Fuel Source	Chemical fuels toxic in vivo	Transition to magnetic, acoustic, or light propulsion
Scalability	Low-yield fabrication	Adoption of nano-/micro-3D printing for reproducible mass production
Navigation and Imaging	Poor visualization in vivo	Integration with MRI or ultrasound tracking
Regulation	Undefined safety standards	Early engagement with FDA/EMA for hybrid device–drug frameworks
Biofilm-Targeting Efficiency	Variable penetration and mechanical force	AI-guided swarm control and field optimization

## Data Availability

Supporting data for this manuscript were sourced via systematic searches of Web of Science, Google Scholar, PubMed, and Scopus.
